# SLC3A2 inhibits ferroptosis in laryngeal carcinoma via mTOR pathway

**DOI:** 10.1186/s41065-022-00225-0

**Published:** 2022-01-20

**Authors:** Fangxing Wu, Gaoyun Xiong, Zejun Chen, Chenyang Lei, Qianqian Liu, Yundan Bai

**Affiliations:** 1 Department of Otolaryngology, Tongde Hospital of Zhejiang Province, No. 234 Gu Chui Road, Hangzhou, Zhejiang Province China; 2 Department of General Medicine, Chengdu First People’s Hospital, Chengdu, 610000 Sichuan Province China; 3 Department of Respiratory and Critical Care Medicine, Chengdu First People’s Hospital, Chengdu, 610000 Sichuan Province China; 4 Department of Health Management Medical Center, Chengdu First People’s Hospital, Chengdu, 610000 Sichuan Province China

**Keywords:** SLC3A2, Laryngeal carcinoma, Ferroptosis, mTOR, Cell proliferation

## Abstract

**Objective:**

This study aimed to explore the mRNA and protein expression of SLC3A2 in laryngeal carcinoma cells and tissues, and functional regulatory mechanism of SLC3A2 in cell ferroptosis of laryngeal carcinoma.

**Methods:**

We chose the key gene-SLC3A2 of DEGs from TCGA by bioinformatics analysis, and then we constructed stable knockdown of SLC3A2 in laryngeal carcinoma cells. MTT assay and clonogenic assay were used to determine cell viability and cell growth, respectively. The mRNA and protein expression were determined by RT-qPCR and western blotting, respectively. Xenograft tumor model was used to determine the role of SLC3A2 in tumor growth.

**Results:**

The results of limma analysis recovered that 92 genes were involved in both upregulated DEGs and high risk of poor prognosis, whereas 36 genes were involved in both downregulated DEGs and low risk of poor prognosis. Pathway enrichment analysis indicated that mTOR signaling pathway and ferroptosis exerted a role in regulating these intersection genes. Moreover, SLC3A2 is a key gene in ferroptosis in laryngeal carcinoma. SLC3A2 is highly expressed in laryngeal carcinoma tissues and cells. Patients with high SLC3A2 expression exerted poor survival. SLC3A2 deficiency inhibited cell proliferation and foci formation. Furthermore, knockdown of SLC3A2 expression induced the efficacy of ferroptosis and suppressed ferroptosis related proteins expression. Mechanically, SLC3A2 deficiency facilitated ferroptosis through upregulating the expression of mTOR and P70S6K, whereas inhibited p-mTOR and p-P70S6K expression in laryngeal carcinoma cells. SLC3A2 deficiency inhibited tumorigenesis in nude mice.

**Conclusion:**

Our study suggests that SLC3A2 negatively regulates ferroptosis through mTOR pathway in laryngeal carcinoma.

## Introduction

Ferroptosis is an iron-dependent non-apoptotic cell necrosis, which relies on iron ions and reactive oxygen species to induce lipid peroxidation and lead to regulatory cell necrosis [1–3]. It is used in morphology, biology, and genetics. The level of ferroptosis is significantly different from other forms of regulatory cell necrosis such as apoptosis and necrosis [4–6]. Its essence is the metabolic disorder of lipid oxides in the cell, which is then abnormally metabolized under the catalysis of iron ions, producing a large amount of lipids, destroying the intracellular redox balance, attacking biological macromolecules, and triggering cell death [3]. In recent years, according to a largenumber of literature reports, GPX4 has been identified as a key regulator of iron death [7, 8], but the research on the mechanism that mediates the production of lipid hydroperoxide is still lacking.

SLC3A2 is a molecular chaperone of light chain subunits (such as SLC1A5 and SLC7A11), and can act by regulating its recruitment to the plasma membrane [9, 10]. By regulating amino acid transport, SLC3A2 and SLC7A5 play an important role in tumor growth and oxidative stress control. Due to these functions, the overexpression of SLC3A2 and SLC7A11 is related to the occurrence and development of various types of cancer, including laryngeal carcinoma. Upregulation of SLC7A11 significantly decreased the ferroptosis caused by IMCA. Additionally, the activities of SLC7A11 and ferroptosis were involved in the AMPK/mTOR/p70S6k signaling pathway regulated by IMCA [9]. Zhu and colleagues reported that SLC3A2 was highly expressed in human osteosarcoma and facilitated tumor growth via the PI3K/Akt signaling pathway [11]. Moreover, the interaction of CD98hc (SLC3A2)/integrin was required for adhesion-induced phosphorylation of FAK and activation of PI3k/Akt and MEK/ERK signaling pathways, while upregulation of a constitutive active form of FAK rescued the CD98hc deficiency [12]. The mechanism of SLC3A2 in laryngeal cancer is currently unclear.

In this study, we explored the relationship between SLC3A2 and iron death. The gene SLC3A2 may be related to iron death through pre-life information mining, and analysis shows that it may play a role through the mTOR pathway. We used public databases to investigate the expression of SLC3A2 in cancer tissues and its relationship with the prognosis of cancer patients. We found that the expression of SLC3A2 in cancer tissues is higher than that in normal tissues, and is related to the poor prognosis of cancer patients, which suggests that targeting SLC3A2 may bring clinical benefits in cancer patients. In addition, iron death plays a role in the anti-cancer effects of SLC3A2 in vivo and in vitro. However, it is unclear whether iron death plays a role in the cancer-promoting effects of SLC3A2.

## Results

### Screening of DEGs associated with prognosis in laryngeal carcinoma

**Fig. 1:**
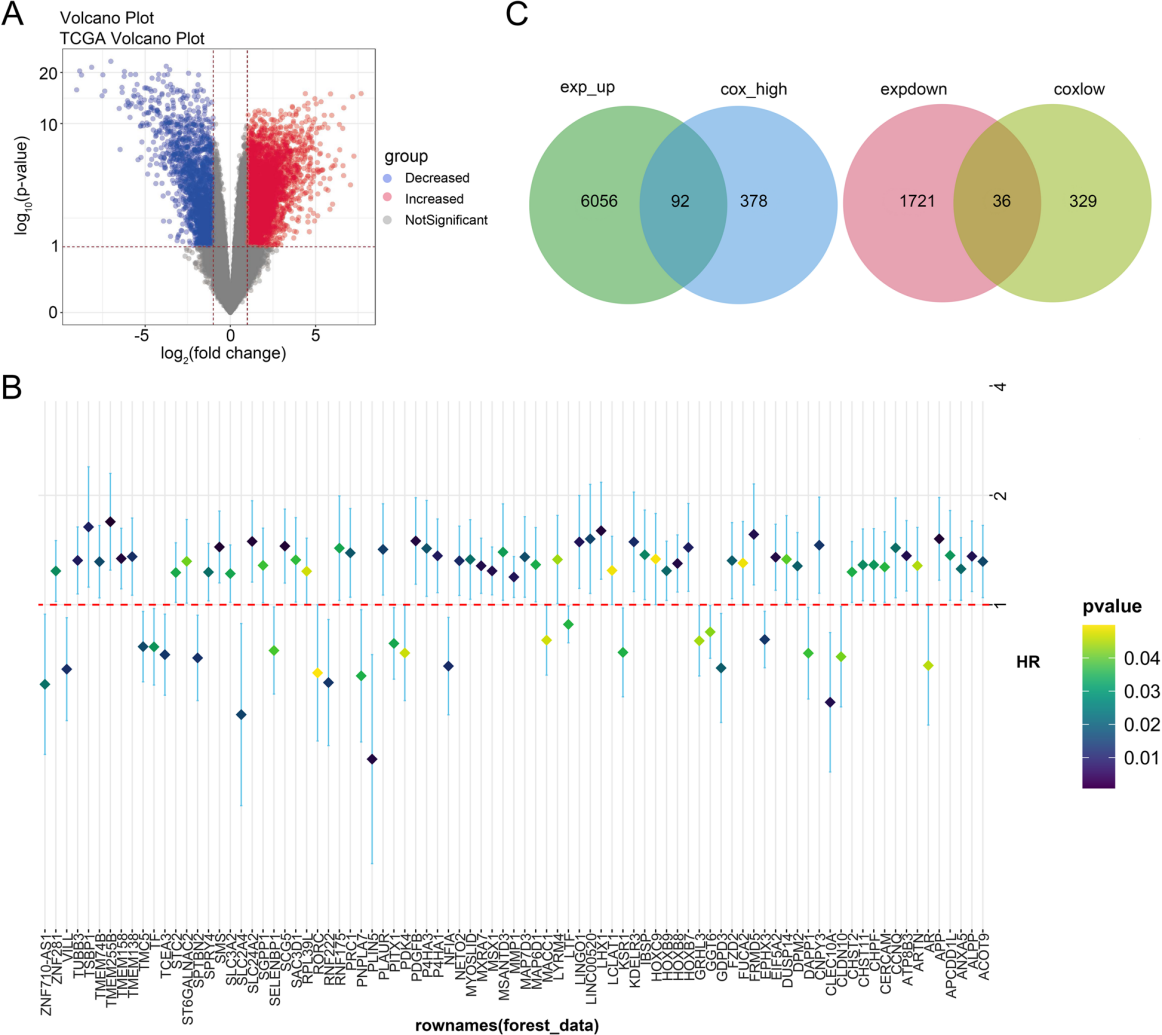
Screening of DEGs associated with prognosis in laryngeal carcinoma. **A** TCGA transcriptome data analysis of significantly expressed differential genes in laryngeal cancer. Blue: Decreased expression genes. Red: Increased expression genes. Gray: Not significant change genes. **B** Single factor cox regression analysis of DEGs involved in prognosis of laryngeal cancer. **C** DEGs and prognosis related genes were analyzed by venn plot

**Fig. 2:**
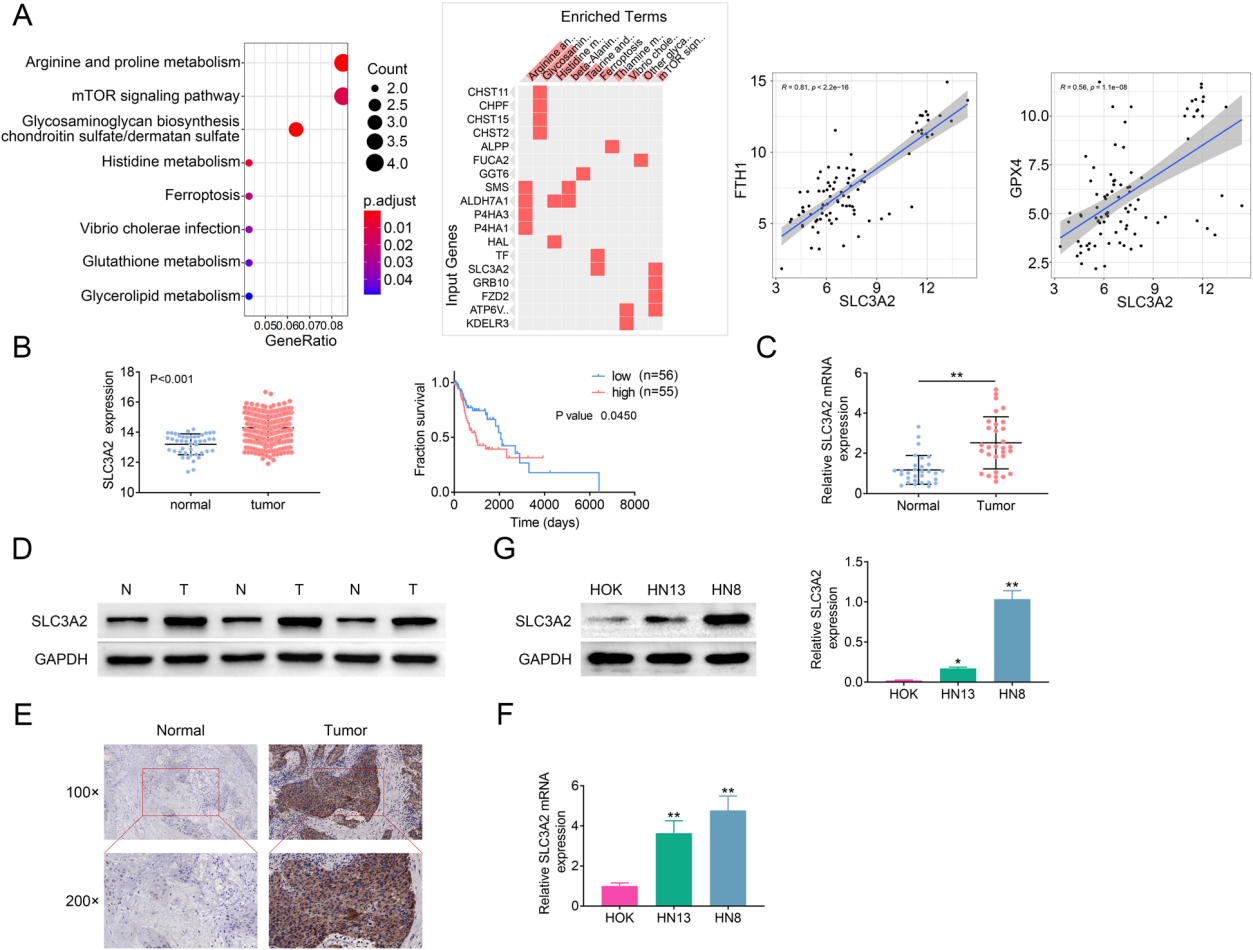
SLC3A2 is a key gene related to ferroptosis in laryngeal carcinoma. **A** Progression related DEGs were analyzed by KEGG-GO and enrichment. **B** Expression (*p* < 0.001) and prognosis (*p* < 0.045) of SLC3A2 between normal and tumor specimens in the database from TCGA. **C** RT-qPCR analysis the expression of SLC3A2 mRNA in normal (*n* = 30) and tumor (*n* = 30) specimens. **D** The expression of SLC3A2 protein in normal and tumor specimens was measured by western blotting. N: normal tissues. T: tumor tissues. *n* = 30. **E** IHC analysis of the expression of SLC3A2 in normal (*n* = 30) and tumor (*n* = 30) specimens. **F**&**G** The mRNA and protein expression of SLC3A2 in normal laryngeal epithelial cells and laryngeal carcinoma cells was measured by RT-qPCR and western blotting, respectively. Error bars represent data from three independent experiments (mean ± SD). *p < 0.05,**p < 0.01

First of all, we analyzed the different expression genes in TCGA-NHSC data (51 normal samples and 495 tumors samples) through limma (Fig. 1A). There is no specific data for laryngeal cancer in TCGA, but laryngeal cancer belongs to head and neck cancer, so TCGA-NHSC data was used. We used R language to analyze the cox survival risk factors of TCGA expression data combined with clinical survival data, we screened genes associated with prognosis of laryngeal carcinoma through univariate cox regression analysis (Fig. 1B). Next, we selected intersection genes both in DEGs and prognosis related genes. As shown in Fig. 1C, 92 genes are both increased DEGs and high risk genes by cox analysis, while 36 genes are both downregulated DEGs and low risk genes by cox analysis. We further analyzed these intersection genes by function and pathway enrichment analysis. As shown in Fig. 2A, most of these genes were involved in mTOR signaling pathway and ferroptosis. Additionally, SLC3A2 was a key gene involving in ferrroptosis, and SLC3A2 exerted significantly positive correlation with FTH1(R = 0.81, *p* < 2.2E-15) and GPX4 (R = 0.56, *p* = 1.1E-6).

### SLC3A2 is a key gene related to ferroptosis in laryngeal carcinoma

In order to clarify the role of SLC3A2 in laryngeal carcinoma, we first analyzed the expression of SLC3A2 in TCGA dataset. As shown in Fig. 2B, SLC3A2 was highly expressed in laryngeal carcinoma compared with normal (*p* < 0.001), and patients with high SLC3A2 expression showed a poor fraction survival (*p* = 0.045). Furthermore, we analyzed the mRNA and protein expression of SLC3A2 in laryngeal carcinoma tissues and cells. As shown in Fig. 2C and D, SLC3A2 was overexpressed in tumor tissues compared with normal tissues. The expression of SLC3A2 was also upregulated in laryngeal carcinoma cells (HN13 and HN8) compared with normal cells (HOK) (Fig. 2G and F). In addition, SLC3A2 was highly expressed in tumor compared with matched normal tissues by IHC (Fig. 2E). Taken together, our data indicated that SLC3A2 is overexpressed in laryngeal carcinoma and associated with poor survival.

### SLC3A2 deficiency decreases cell proliferation and foci formation

**Fig. 3:**
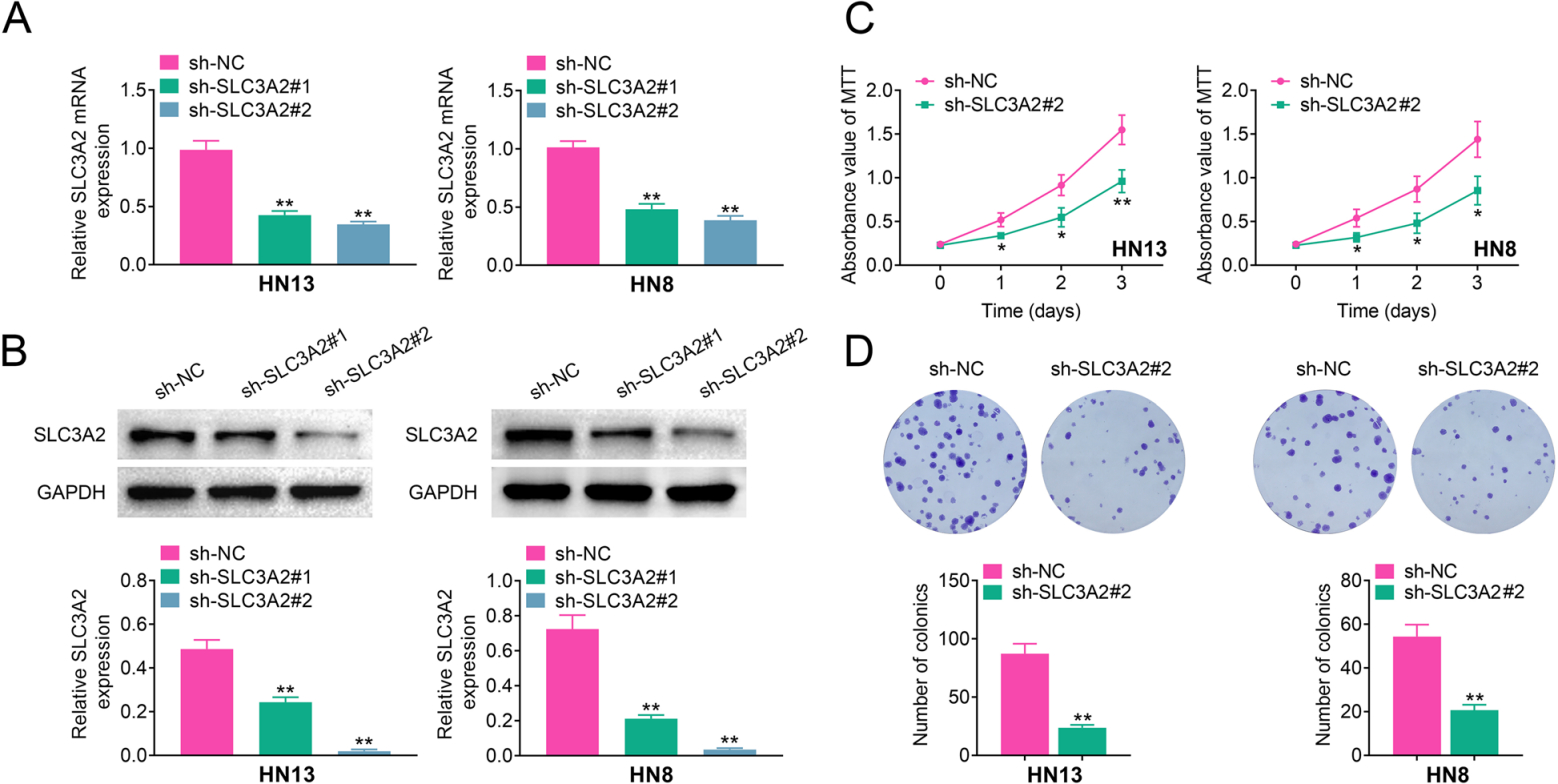
SLC3A2 deficiency decreases cell proliferation. **A** HN13 and HN8 cells were transfected with indicated plasmids, and the mRNA expression of SLC3A2 was determined by RT-qPCR. **B** The expression of SLC3A2 protein was determined by western blotting. **C** The cell proliferation in SLC3A2 knockdown HN13 and HN8 cells was tested by the MTT assay. **D** Clonogenic assay was performed to measure the capacity of foci formation in knockdown of SLC3A2 in HN13 and HN8 cells. Error bars represent data from three independent experiments (mean ± SD). *p < 0.05,**p < 0.01

Next, we constructed stable knockdown of SLC3A2 in HN13 and HN18 cells, measured by RT-qPCR and western blotting (Fig. 3A&3B). Then we examined the cell viability in SLC3A2 knockdown laryngeal carcinoma cell lines by MTT assay. As shown in Fig. 3C, knockdown of SLC3A2 in HN13 and HN8 cell lines suppressed cell proliferation compared with matched sh-NC cell lines. Furthermore, SLC3A2 deficiency inhibited colony formation in HN13 and HN8 cell lines (Fig. 3D). Collectively, our data suggested that SLC3A2 positively regulates cell viability and foci formation in laryngeal carcinoma cells.

### SLC3A2 deficiency induces ferroptosis in laryngeal cancer cells

**Fig. 4:**
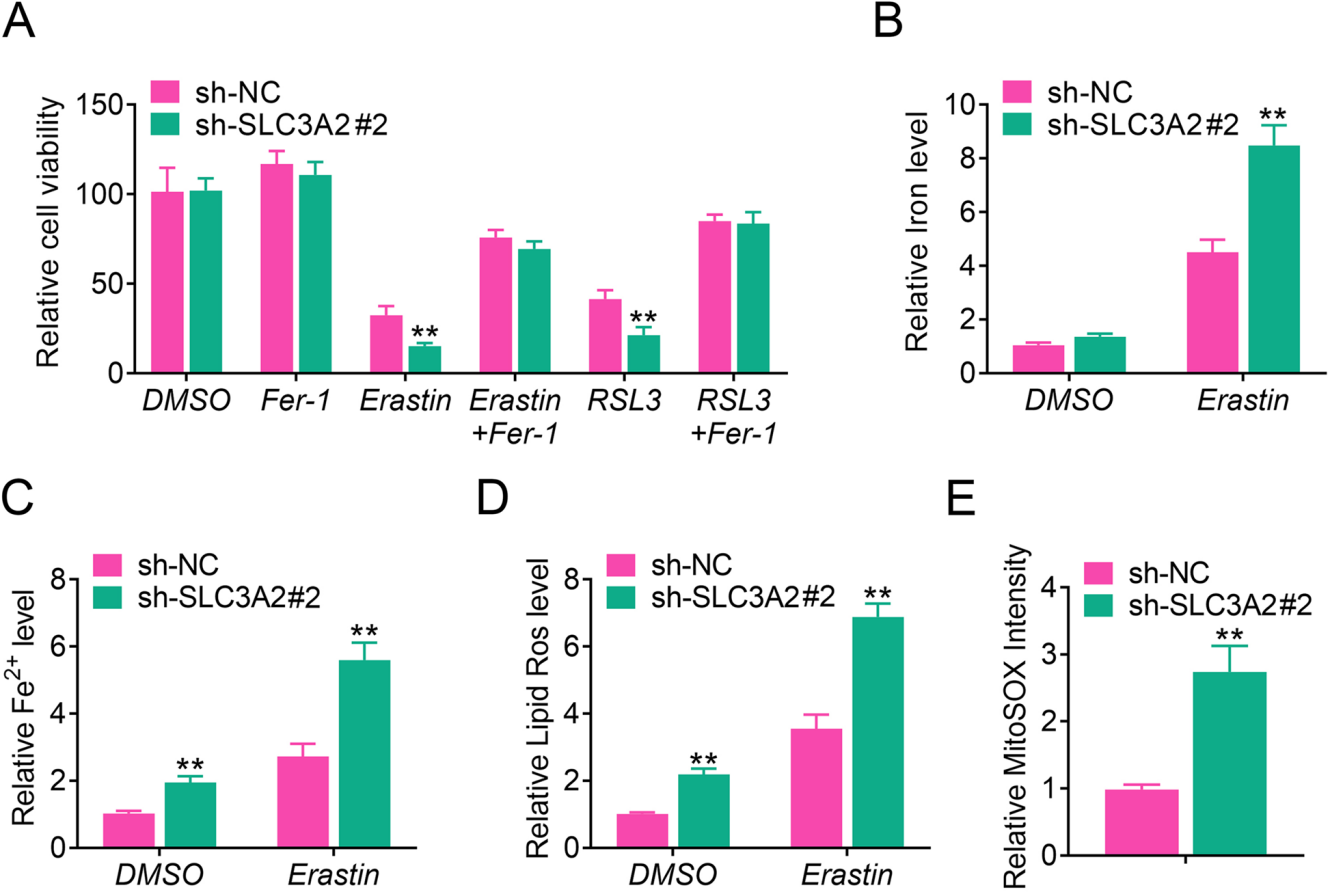
SLC3A2 deficiency induces ferroptosis in HN13. **A** HN13-shNC and shSLC3A2 cells were treated with DMSO, Fer-1 (2.0 μM), Erastin (10.0 μM), Fer-1 (2.0 μM) + Erastin (2.0 μM), RSL3 (2.0 μM) and Fer-1 (2.0 μM) + RSL3 (2.0 μM), respectively, for 48 h and then cell viability was evaluated by MTT assay. In order to better show the difference of cell viability in different treated groups, we converted the cell viability of control group (treated with DMSO) to 100%, and then compare the cell viability of other groups. **B**&**C** The levels of total iron level and Fe^2+^ in laryngeal cancer cells were determined using iron assay kit. **D** The level of lipid ROS in laryngeal cancer cells was analyzed using cellular ROS assay kit. **E** The production of mitochondrial superoxide in laryngeal cancer cells was examined using MitoSOX™ Red Mitochondrial Superoxide Indicator. Results presented represent the means of triplicate experiments ± SEM. **p < 0.01

As shown in Fig. 2A, SLC3A2 was found to be a key genes involved in ferroptosis. Therefore, we next analyzed the role of SLC3A2 in ferroptosis of laryngeal carcinoma. As shown in Fig. 4A, cell viability was significantly suppressed in SLC3A2 knockdown HN13 cells treated with erastin, or RSL3 + Fer-1 compared with si-NC group cell lines. Next, we analyzed the effect of SLC3A2 on regulating of iron and Fe^2+^. As shown in Fig. 4B and C, knockdown of SLC3A2 significantly increased iron and Fe^2+^ level in HN13 cell lines treated with erastin. Furthermore, the levels of lipid ROS and mitochondrial superoxide were also upregulated in SLC3A2-knockdown HN13 cell lines treated with erastin (Fig. 4D&4E). Taken together, our data indicated that SLC3A2 is a suppressor in regulating of ferroptosis in laryngeal carcinoma cells.

### SLC3A2 inhibits ferroptosis partly through mTOR pathway

**Fig. 5:**
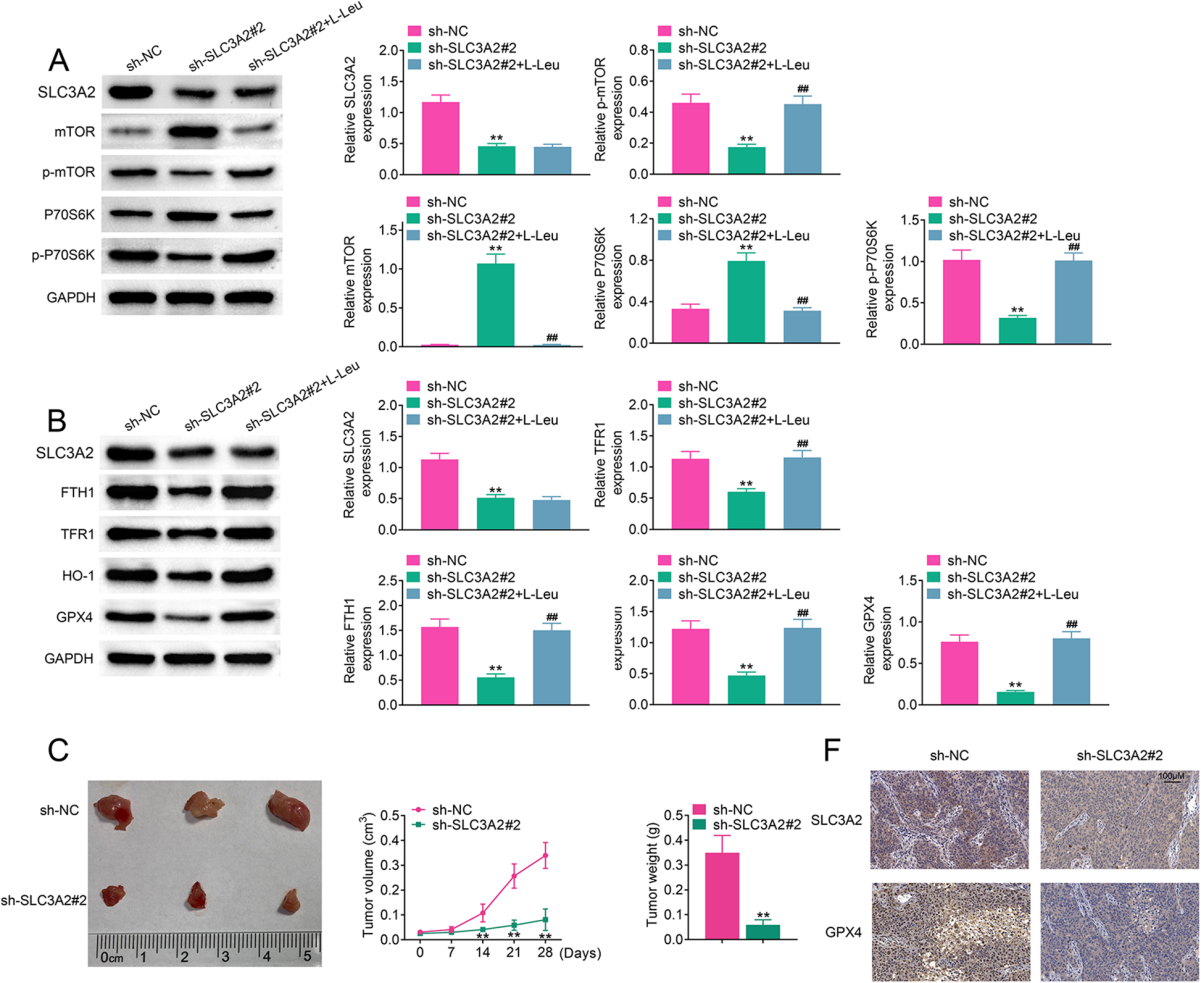
SLC3A2 inhibits ferroptosis partly through mTOR pathway. **A** SLC3A2-Knockdown laryngeal cancer cells were treated with or without L-Leu (activator of mTOR pathway), the protein expression of mTOR, p-mTOR, P70S6K and p-P70S6K was measured by western blotting. **B**-**F** The same cells were treated as in (A), then western blotting was perform to measure the ferroptosis related protein expression. **G** HN13 cells stably expressing control, SLC3A2 knockdown plasmids were injected subcutaneously into nude mice. Representative images showing xenograft mice tumors at day 28 post subcutaneous injection (*n* = 3). Tumor sizes were measured and depicted as tumor volume and tumor weight. Results presented represent the means of triplicate experiments ± SEM. **p < 0.01. (H) IHC analysis of SLC3A2 and GPX4 expression in xenograft mice tumors. Scale bars, 100 μm

Next, we clarified its underlying mechanism of SLC3A2 in ferroptosis of laryngeal carcinoma cells. First of all, we analyzed the mTOR pathway proteins expression in SLC3A2-knockdown cell lines. As shown in Fig. 5A, SLC3A2 deficiency upregulated the expression of mTOR and P70S6K, whereas inhibited p-mTOR and p-P70S6K expression in laryngeal carcinoma cells. SLC3A2-knockdown HN13 cell lines treated with L-Leu antagonized the inhibition of p-mTOR and p-P70S6K expression. Ferroptosis was an iron-dependent lipid peroxidation process associated with the inhibition of glutathione peroxidase 4 (GPX4) activity. Ferroptosis was also a form of autophagic cell death involving the degradation of ferritin, involving ferritin heavy chain 1 (FTH1). Therefore, we also analyzed the ferroptosis related proteins expression. As shown in Fig. 5B-5F, SLC3A2 deficiency decreased the expression of FTH1, TFR1, HO-1, and GPX4, whereas SLC3A2-knockdown HN13 cell lines treated with L-Leu antagonized the suppression of these protein expression. Additionally, SLC3A2 deficiency suppressed tumor growth in nude mice (Fig. 5G), and the expression of GPX4 was upregulated in sh-SLC3A2 tumor tissues compared to sh-NC tumor tissues by IHC staining. Collectively, our data suggested that SLC3A2 negatively regulates ferroptosis partly through mTOR pathway.

## Discussion

Laryngeal carcinoma is still one of the most common tumors of the respiratory tract. Fortunately, in the past ten years, the treatment of laryngeal cancer has made significant progress. Although surgery has always been the main treatment for localized diseases, and it is still an important part of treatment, non-surgical methods such as radiotherapy and systemic treatment have become viable options. In addition, in a metastatic environment, new drugs show promise for this patient group. Therefore, it is essential to find new target for laryngeal carcinoma treatment. Here, we screened out SLC3A2 as a key gene involved in ferroptosis in laryngeal carcinoma by TCGA data analysis. SLC3A2 deficiency negatively regulated cell growth and tumorigenesis in laryngeal carcinoma. Previous studies reported that upregulation of SLC3A2 facilitated cell proliferation and was associated with patient survival and progress in oral cancer [13]. Compared with normal breast tissue, the expression of xCT components SLC7A11 and SLC3A2 is increased in a subset of patient breast tumor tissues, and their co-expression is closely related to the expression of GPX4 [14]. SLC3A2 is also important in preventing excessive lipid peroxidation in cells [9, 15]. In addition, SLC3A2 is also necessary for erastin-induced iron drop disease [16].

Based on the functional importance of SLC3A2, it is essential to identify the underlying mechanism that may mediate ferroptosis. In this study, we demonstrated that SLC3A2 as a key component involving ferroptosis, is upregulated in laryngeal carcinoma. Previous study reported that SLC3A2 is associated with poor prognostic characteristics and poor survival outcome in breast cancer [17]. Recently, overexpression of SLC3A2 was associated with poor survival of OSCC patients [13]. High SLC3A2 expression is associated with poor survival in laryngeal carcinoma, though there is a reversely result that some patients with higher SLC3A2 displayed longer survival after 3000d. In the middle and late stages, other complications and other factors may affect the survival rate. For example, SLC3A2-NRG1 formed a fusion gene positive regulation of lung cancer [18]. Additionally, SLC3A2 was a target gene of ZEB1 in breast cancer chemoresistance, it maybe the chemotherapy of laryngeal carcinoma is associsated with the result of high SLC3A2 patients with longer survival. We also confirmed that SLC3A2 negatively regulates cell viability, migration and invasion. In addition, we demonstrated that SLC3A2 deficiency decelerates cell ferroptosis of laryngeal carcinoma. Moreover, we demonstrated that SLC3A2 deficiency decreases tumor growth in vivo. Ferroptosis originally served as a unique form of cell death induced by small molecules, such as Fer-1, erastin and RSL3, to screen for their selective cytotoxicity in cells overexpressing the oncogenic mutant HRAS [19, 20]. Morphologically, cell ferroptosis has a characteristic necrotic morphology, accompanied by dysmorphic small mitochondria with decreased crista, a condensed membrane, and a ruptured outer membrane [21]. Our study also used erastin and RSL3 to clarify the function of SLC3A2 in ferroptosis, and we demonstrated that SLC3A2 deficiency inhibits ferroptosis. Furthermore, SLC3A2 was associated with GPX4 and mTOR signal pathway by enrichment analysis.

Recently, the selenium enzyme glutathione peroxidase 4 (GPX4) was identified as a key enzyme that uses glutathione to reduce lipid hydroperoxides to lipid alcohols, thereby protecting cells from iron drop disease [22, 23]. Unlike other GPXs, GPX4 is the only enzyme found to act on hydrogen peroxide phospholipids [22]. Under physiological conditions, GPX4 reduces lipid hydrogen peroxide, but at the expense of reducing the form of its cofactor glutathione [23]. In addition to the results of SLC3A2 mediated iron drop through the SLC3A2/mTOR axis, we also further analyzed the expression of GPX4 in SLC3A2-deficient tumors. Western blotting results showed that GPX4 is positively regulated by SLC3A2. But its molecular function regulation mechanism needs further study.

Previous study demonstrated that SLC7A5 functions as a glycoprotein-related heterodimer together with the multifunctional protein SLC3A2, SLC7A5 modulates LNAA-dependent muscle mTOR-S6K signaling in mice. Therefore SLC3A2 may also act as an important component in tumorigenesis through mTOR-S6K signal pathway [24]. SLC7A11 overexpression significantly decreased the ferroptosis caused by IMCA. Additionally, the activities of SLC7A11 and ferroptosis were involved in the AMPK/mTOR/p70S6k signaling pathway regulated through IMCA [9]. SLC7A11 negatively regulates the Nrf2/HO-1 signal. Nrf2 and HO-1 are activated in this model due to the accumulation of intracellular ROS. Up-regulation of SLC7A11 can increase the level of intracellular cystine, reduce the oxidative stress of epithelial cells, and inhibit the positive stimulation of Nrf2/HO-1 expression to a certain extent [25]. SLC3A2 as a co-component chain with SLC7A11 in System x(c)(−) cystine/glutamate antiporter, may exert similar role in ferroptosis involved in the ROS and mTOR signaling pathway. The SLC3A2-NRG1 fusion induced phosphorylation of ERBB2-ERBB3 and ectopic duplex formation, and activated the downstream PI3K/AKT/mTOR pathway through paracrine signals [26]. Overactivity of PI3K-AKT-mTOR signaling mutation protected cancer cells from oxidative stress and iron-related death through SREBP1/SCD1-mediated adipogenesis [27]. By promoting AMPK-mTOR pathway to up-regulate the enhanced autophagy activation of sirt3, reduced the level of GPX4, and induced ferroptosis in trophoblast cells. SIRT3 deficiency was resistant to autophagy-dependent ferroptosis induced by high glucose and villastine [28]. In addition, the interaction of rapamycin kinase (mTOR) mechanism target signal and glutathione peroxidase 4 (GPX4) signal can regulate autophagy-dependent ferroptosis in human pancreatic cancer cells [29]. Therefore, we similarly measured mTOR expression in SLC3A2 deficiency cells. Next, we consider to confirm whether SLC3A2 directly regulates mTOR in laryngeal cancer.

## Conclusions

Our study suggests that SLC3A2 regulates ferroptosis and related genes, and therefore subsequently induces laryngeal carcinoma growth and ferroptosis via mTOR signal pathway. Since ferroptosis can be negatively regulated by SLC3A2/mTOR axis, our results suggest that targeting SLC3A2 may be an underlying approach for laryngeal tumor therapy.

## Materials and methods

### Cells, tissues and nude mice

HOK, HN13 and HN8 cells were purchased from the Cell Bank of the Chinese Academy of Sciences (Shanghai, China) and cultured in Dulbecco’s modified Eagle’s medium (DMEM) supplemented with 10% FBS and 100 U/ml penicillin/streptomycin. All the cell lines were maintained in 37 °C, 5% CO_2_ incubator. The experimental manipulations performed have been previously reported [30, 31]. All human specimens were collected and preserved as described previously (Table 1) [32]. All procedures performed in studies involving human participants were in accordance with the standards upheld by the Ethics Committee of Tongde Hospital of Zhejiang Province (Approval no. 2016003) and with those of the 1964 Helsinki Declaration and its later amendments for ethical research involving human subjects. All animal experiments were approved by the Animal Welfare Ethics Committee of Zhejiang Academy of Traditional Chinese Medicine (Approval no. KTSC2020390) for the use of animals and conducted in accordance with the National Institutes of Health Laboratory Animal Care and Use Guidelines.

**Table 1:** Baseline data of clinical patients. 30 specimens were used in the research. All specimens were divided into two groups dependent of the expression of SLC3A2. In addition, the specimens were also classification of gender, age, pathological stage and grade

	Low_exp	High_exp	*P*-value
Total	19	11	/
Gender			Chi square test
male	10	5	*p* > 0.05
female	9	6	
Age			*p* > 0.05
< 65	6	4	
> =65	13	7	
Stage			*P* = 0.030
I	8	1	
II	6	1	
III	3	4	
IV	2	5	
Grade			*P* = 0.049
1	7	1	
2	8	2	
3	2	4	
4	2	4	

### Cell viability and colony formation assays

The Cell proliferation and viability assay were performed as described previously [33]. 1 × 10^4^ cells were seeded in a 96-well-plate in triplicate and the colorimetric MTT Assay kit (keygenbio) was used to monitor the cell viability rate until 3 days. For the clonogenic assay, 1 × 10^¬3^ cells were seeded in 6-well plates to form colonies. After 72 h, colonies were stained, photographed, and scored. Colonies with no fewer than 50 cells per colony were counted.

### Immunohistochemical assay

The immunohistochemical assay was performed as described previously [34]. Tumor sections were deparaffinized, blocked, incubated, and counterstained. Staining results were examined by light microscopy for histological changes.

### RNA isolation and real-time quantitation PCR

Total RNA was extracted using TRIzol reagent (Ambion, CA, USA). A total of 1 μg of RNA was reverse-transcribed using the ImProm-IITM Reverse Transcription System (Promega, WI, USA). Quantitative real-time RT-PCR was conducted using SYBR GREEN qPCR Super Mix (Invitrogen, CA, USA). A standard amplification protocol was used according to the supplier’s directions. Primers were listed as following.

- SLC3A2 forward: 5′-TGAATGAGTTAGAGCCCGAGA -3′;
- SLC3A2 reverse: 5′-GTCTTCCGCCACCTTGATCTT -3′;
- GAPDH forward: 5′- AGACAGCCGCATCTTCTTGT-3′;
- GAPDH reverse: 5′- CTTGCCGTGGGTAGAGTCAT-3′;

### Western blotting

Western blotting was performed as described as previously depicted [15]. And immunoblotted with the following antibodies: anti-mouse SLC3A2 (1:1000, Santa, sc-376,815, USA), anti-mouse mTOR (1:1000, Santa, sc-517,464, USA), anti-mouse p-mTOR (1:1000, Santa, sc-293,133, USA), anti-mouse P70S6K (1:1000, Santa, sc-8418, USA), anti-mouse p-P70S6K (1:1000, Santa, sc-8416, USA), anti-mouse FTH1 (1:1000, Santa, sc-376,594, USA), anti-mouse TFR1 (1:1000, Santa, sc-32,272, USA), anti-Rabbit HO-1 (1:1000, abcam, ab52947, England), anti-mouse GPX4 (1:1000, Santa, sc-166,120, USA), and anti-mouse GAPDH (1:1000, Santa, sc-47,724, USA). Then, the PVDF membranes were washed and secondary antibodies were applied 1:5000 for 1 h at room temperature, the immunoreactions were visualized with chemiluminescent ECL reagent.

### Iron, lipid peroxide ROS, mitochondrial membrane potential detection

The levels of total iron level and Fe^2+^ in HCC cells were determined using Iron Assay Kit (ab83366; Abcam). The level of lipid ROS in HCC cells was analyzed using Cellular ROS Assay Kit (ab186029; Abcam) based on the protocols of anufacturers. Mitochondrial superoxide assay: The production of mitochondrial superoxide in HCC cells was examined using MitoSOX™ Red Mitochondrial Superoxide Indicator, for live-cell imaging (Thermo Fisher Scientific). Briefly, 5 mM MitoSOX™ reagent stock solution was diluted in Hank’s buffered salt solution (HBSS)/Ca/Mg buffer to make a 5 μM MitoSOX reagent working solution. Then 2 mL working solution was used to cover HCC cells adhering to coverslips. Cells were maintained in the dark for 10 min at 37 °C. Thereafter, phosphate-buffered saline (PBS; Solarbio) was used to wash cells.

### Nude mice tumorigenesis in vivo assay

In vivo tumorigenesis assays were conducted as previously described [34]. The tumor volume was calculated as follows: (length × width^2^)/2. After 24 days, the mice were killed and their tumors were collected, fixed and sectioned, stained by hematoxylin and eosin, and examined by light microscopy for histological changes.

### Volcano plot, Kaplan-Meier, and GO-KEGG analysis

The RNA sequencing profiles were downloaded from TCGA. All the data were public and available. Different expression genes from TCGA were determined by bioinformatical analysis (limma analysis different expression genes, data filtering and standardization were performed as described previously [35]. Processing of TCGA data was performed as described as previously depicted, including download method (TCGAbiolinks) [36, 37], filtering conditions [37, 38]. The cut-off criterion was set as FDR < 0.05 & |logFC| > 1. Venn diagram tool (http://bioinfogp.cnb.csic.es/tools/venny/) was then used to identify the overlapping genes of DEGs analyzed by edgeR and limma package. After preprocessing the data from TCGA, extract TPM type tumor expression data. According to the expression level of SLC3A2, high and low groups (median) were made. The results of differentially expressed genes analysis performed by limma and edgeR, and the previous (normal vs tumor) differential analysis results were subjected to venn analysis to obtain genes that are highly correlated with SLC3A2 expression and have clinical significance, and perform KEGG-GO analysis by using of the clusterProfiler package for R language [39, 40]. For survival analysis, the prognosis of patient is measured by online software Kaplan-Meier curve tool (http://kmplot.com/analysis/).

### Statistical analysis

Statistical analyses were performed using One-way ANOVA, Newman-Keuls Multiple Comparison Test by Graphpad prism 5 (Graphpad Software, San Diego, CA). Data were presented as means ± SEM of at least three independent experiments. A *P*-value of 0.05 or less were considered to be statistically significant.

## Data Availability

The data supporting the findings of the article is all available in the this published article.

## Abbreviations

GPX4: Glutathione peroxidase 4
DMEM: Dulbecco’s modified Eagle’s medium
HBSS: Hank’s buffered salt solution
